# Independent association between socioeconomic indicators and macro- and micro-nutrient intake in Switzerland

**DOI:** 10.1371/journal.pone.0174578

**Published:** 2017-04-03

**Authors:** Carlos de Mestral, Pedro Marques-Vidal, Jean-Michel Gaspoz, Jean-Marc Theler, Idris Guessous

**Affiliations:** 1 Institute of Social and Preventive Medicine, Lausanne University Hospital, Lausanne, Switzerland; 2 Department of Internal Medicine, Internal Medicine, Lausanne University Hospital, Lausanne, Switzerland; 3 Unit of Population Epidemiology, Division of Primary Care Medicine, Department of Community Medicine, Primary Care and Emergency Medicine, Geneva University Hospitals, Geneva, Switzerland; 4 Department of Epidemiology, Rollins School of Public Health, Emory University, Atlanta, United States of America; University of Padova, ITALY

## Abstract

**Background:**

Socioeconomic differences in diet are rarely assessed with more than one indicator. We aimed to assess differences in macro- and micro-nutrient intake in both sexes according to education, income, and occupation.

**Methods:**

We used data from validated food frequency questionnaire measured dietary intake in 5087 participants (2157 women) from yearly adult population-based cross-sectional surveys conducted from 2005 to 2012 in the canton of Geneva, Switzerland. We used two ANOVA models: age-adjusted and multivariable adjusted simultaneously for all three socioeconomic indicators.

**Results:**

Low-education men consumed more calcium but less vitamin D than high-education men; low-income men consumed less total and animal protein (80.9±0.9 vs 84.0±0.6 g/d; 55.6±1.0 vs 59.5±0.7 g/d) and more total carbohydrates and sugars (246±2 vs 235±2 g/d; 108±2 vs 103±1 g/d) than high-income men. Occupation and diet showed no association. Low-education women consumed less vegetable protein (20.7±0.2 vs 21.6±0.2 g/d), fibre (15.7±0.3 vs 16.8±0.2 g/d), and carotene (4222±158 vs 4870±128 μg/d) than high-education women; low-income women consumed more total carbohydrates (206±2 vs 197±1 g/d) and less monounsaturated fat (27.7±0.4 vs 29.3±0.3 g/d) than high-income women. Finally, low-occupation women consumed more total energy (1792±27 vs 1714±15 kcal/d) and total carbohydrates (206±2 vs 200±1 g/d), but less saturated fat (23.0±0.3 vs 24.4±0.2 g/d), calcium (935±17 vs 997±10 mg/d) and vitamin D (2.5±0.1 vs 2.9±0.1 μg/d), than high-occupation women.

**Conclusion:**

In Switzerland, the influence of socioeconomic factors on nutrient intake differs by sex; income and education, but not occupation, drive differences among men; among women, all three indicators seem to play a role. Interventions to reduce inequalities should consider the influence of education, income, and occupation in diet to be most effective.

## Introduction

In high-income countries, socioeconomic status (SES) determines nutritional quality—people of low SES tend to follow unhealthier diets compared to people of high SES [1, 2]. Unhealthier diets contribute to the development of obesity, hypertension, dyslipidemia and diabetes, and increase the risk of cardiovascular disease (CVD) and certain cancers [3, 4]. People of low SES tend to be disproportionally affected by these risk factors and morbidities [5, 6]. It is thus imperative to assess the associations between SES and nutrition, but the literature remains scarce in assessing the associations between dietary intake and SES with more than one SES indicator—most reports evaluate only one SES indicator, most often education [1, 2]. However, different indicators—education, income, occupation—relate to different underlying social processes that likely influence dietary intake differently and should not be used interchangeably [1, 7]; education likely influences understanding of nutrition labels and reception of public health preventive messages; income likely impacts food purchasing behaviour; and occupation likely affects eating behaviour [1, 7]. Assessing the independent association of each indicator on diet would enable to create specific and better informed public health interventions likely to be more effective. Recent randomized controlled trials suggested that price discounts might be more important than education in promoting healthier food purchasing behaviour [8, 9], but whether this also applies to the general population has seldom been assessed.

Switzerland is a small European country with relatively low CVD mortality rates [10], but overweight and obesity rates have increased significantly over the past decades, with varying trends by SES [11, 12]. Previous findings in the French-speaking canton of Geneva revealed that from 1993 to 2000 men and women in high SES—defined by education and occupation—followed healthier diets [13], while those in low SES followed increasingly unhealthier diets [14]. In a previous paper, we assessed the association between one measure of SES—education—and dietary intake [15]. Still, no information is available regarding the associations between dietary intake and other SES indicators, namely income and occupation. Thus, we have now extended our analysis to assess differences in macro and micronutrient intake in both sexes according to education, income, and occupation using data collected between 2005 and 2012 in Geneva.

## Methods

### Study design and sampling

The “Bus Santé” study is a cross-sectional, ongoing, population-based study aiming to collect information on chronic disease risk factors in the canton of Geneva, Switzerland. Its sampling methodology has been previously reported [16]. Since 1993, on a yearly basis, it recruits a representative sample of non-institutionalized men and women aged 35 to 74 years. Eligible participants are identified with a standardized procedure using a residential list established annually by the local government. Random sampling in age- and sex-specific strata matches the corresponding frequencies in the population. Potential participants unreachable upon three mailings and seven phone calls are replaced using the aforementioned protocol, but subjects reached and unwilling to participate are not replaced. Included participants become ineligible for future surveys. Participation rates ranged from 55 to 65%.

### Ethics statement

The “Bus Santé” Geneva study was approved by the University of Geneva ethics committee and all study participants provided informed written consent to participate in the study. The study has been performed in accordance with the ethical standards established in the 1964 Declaration of Helsinki and its later amendments.

### Data collection

Two clinics and one mobile medical unit conducted health examinations from January to December each year. Body weight and height were measured using standard procedures, and BMI (kg/m^2^) was calculated. Self-administered questionnaires collected information on socio-demographic characteristics, smoking, education, income, and occupation. Trained collaborators performed the examinations, interviewed participants, and checked for completion of the self-administered questionnaires. Procedures are regularly reviewed and standardized across collaborators.

Participants self-reported their smoking status (never, previous, or current) and country of birth (categorized as Switzerland or other in our analysis). Three measures acted as SES proxies: education, categorized as low (primary education or apprenticeship), medium (secondary education), or high (tertiary education); household income in Swiss francs (CHF)/month (1 CHF = 1.01 USD or 0.91 EUR as of 24.02.2016), categorized as low (<5000 CHF), medium (5000 to 9499 CHF), or high (≥9500 CHF); and occupation, categorized as low (blue collar: manual skilled or unskilled work) or high (white collar: managerial, professional, non-manual work). For participants of retirement age, occupation was determined using last held occupation.

### Dietary intake

Dietary intake during the four preceding weeks was assessed by a semi-quantitative food frequency questionnaire (FFQ) developed and validated in the target population [17]. To our knowledge, there exists no validated FFQ assessing annual dietary intake in Switzerland, so the FFQ used in this study constitutes the best available option to assess dietary intake in the French-speaking Swiss population. The FFQ listed 80 food items divided in 12 food groups accounting for over 90% of energy, proteins, fats, carbohydrates, vitamin D and retinol intake; 85% of fibre, carotene, and iron intake; and 62% of calcium intake. Consumption frequencies ranged from “less than once during the last weeks” to “2 or more times per day,” and serving sizes consisted in smaller, equal or larger than a reference size. A trained interviewer checked each FFQ for completion during the clinic and mobile unit visit. Throughout the study period, the same food composition table converted measured foods to macro and micronutrient intakes. We included all available macro and micronutrients.

### Exclusion criteria

Participants were excluded if they failed to report age, sex, weight, height, smoking, education, income, or occupation or if they reported extreme energy intakes (<850 or >4500 kcal/day) as suggested elsewhere [18].

### Statistical analyses

We used data from 2005 to 2012. Statistical analyses were performed separately for men and women using Stata version 13.1 for Windows (Stata Corp, College Station, Texas, USA). No interaction between sex and SES was found but due to large absolute differences in nutrient intake (i.e. ~400 kcal/day), we decided to stratify the analysis by sex to facilitate results interpretation. Compared to other years, sample sizes from 2005 to 2008 were smaller than subsequent years because another cohort study was conducted concurrently relying on the same infrastructure. Therefore, we combined these years into two groups: 2005–06 and 2007–08. Descriptive results are expressed as number of participants (percentage) or as mean (standard deviation). Bivariate analyses were performed using student’s t-test for continuous variables and chi-square for bivariate or categorical variables.

We formulated no specific hypotheses regarding the association between SES markers and dietary intake. Importantly, we found no significant time trends, SES*time interactions or interactions between the three SES indicators for any nutrients (results provided upon request). We attempted to assess collinearity between SES indicators using Kappa and Spearman statistics, finding only a weak correlation between education and income (Spearman’s rho: 0.27, p<0.001). Multivariable analysis was conducted using ANOVA and two different models: 1) adjusted for age and total energy intake (TEI, including calories from alcohol), and 2) adjusted for age, survey year, BMI, smoking status, nationality and TEI. Linear trends were assessed using the contrast p. option in Stata. We present results for nutrients in g/d, mg/d or μg/d to facilitate comparison between SES groups. Sensitivity analysis was conducted including all previously excluded participants; results are presented in supplementary material. Due to the large number of tests performed, we considered statistical significance for a two-sided test at p<0.01.

## Results

### Selection of participants

From the initial 5087 participants, 658 (12.9%) were excluded (**Figure A in S1 File**). **Table A in S1 File** compares the characteristics of included and excluded participants. Excluded participants were more likely to be women, have medium educational level, low income, and low occupation than included participants.

### Characteristics of included participants

**Table 1**summarizes the characteristics of participants included in the analysis by survey year. From 2005 to 2012, the percentage of participants with high education or high occupation increased significantly. The percentage of participants with high income and of never-smokers also tended to increase, but the trend was not significant. Mean age varied by year, though only slightly. No differences across years were found regarding sex, BMI, and nationality.

**Table 1:** Characteristics of included participants from “Bus Santé” study, Geneva, Switzerland, 2005–2012.

	Total	Women	Men	*p*
N	4429	2157	2272	
Age (mean, SD)	51.3 (11.5)	51.4 (11.4)	51.2 (11.6)	0.70
BMI (mean, SD)	25.6 (8.9)	24.5 (8.3)	26.4 (9.5)	**
Nationality (n, %)				
Swiss	3531 (69.4)	1875 (71.3)	1656 (66.9)	
Other	1556 (30.6)	737 (28.2)	819 (33.1)	
BMI categories (n, %)				**
Underwt or normal	2787 (55.3)	1691 (65.5)	1096 (44.7)	
Overweight	1652 (32.8)	621 (24.0)	1031 (42.0)	
Obese	598 (11.9)	271 (10.5)	327 (13.3)	
Smoking status (n, %)				**
Never smoked	2389 (46.9)	1349 (51.7)	1040 (42.0)	
Ex-smoker	1608 (31.6)	737 (28.2)	871 (35.2)	
Smoker	1090 (21.4)	526 (20.1)	564 (22.8)	
Education level (n, %)				**
Low	1495 (29.4)	699 (26.8)	796 (32.2)	
Medium	1402 (27.6)	863 (33.1)	539 (21.8)	
High	2181 (43.0)	1043 (40.0)	1138 (46.0)	
Income level (n, %)				**
Low	1038 (21.9)	597 (25.1)	441 (18.7)	
Medium	1896 (40.0)	998 (42.0)	898 (38.1)	
High	1802 (38.1)	782 (32.9)	1020 (43.2)	
Occupation (n, %)				**
Low	1468 (29.7)	678 (27.2)	790 (32.3)	
High	3475 (70.3)	1822 (72.9)	1653 (67.7)	

Results are expressed as number of included participants (percentage) or mean (standard deviation). Statistical comparison by ANOVA for age and BMI (body mass index), chi-square for sex, smoking, BMI categories, nationality, education, income, and occupation. BMI, body mass index. p-value for difference between survey years. BMI categories, underweight (underwt) or normal, BMI<25 kg/m^2^; overweight, 25≤BMI<30 kg/m^2^ and obese, BMI≥30 kg/m^2^. Education: low, primary education or apprenticeship; medium, secondary education; high, tertiary education. Income: low, <5000 CHF/month (1 CHF = 1.01 USD or 0.91 EUR as of 24.02.2016); medium, 5000 to 9499 CHF/month; high, ≥9500 CHF/month. Occupation: low, blue collar; high, white collar. *p*, p-value; mean values were significantly different across groups:

*P<0.01

**P<0.001.

### Associations between SES indicators and dietary intakes, adjusted for age and TEI

**Tables 2**and **3**summarize the associations between SES indicators and dietary intakes adjusted for age and TEI for men and women, respectively.

**Table 2:** Energy and nutrient intake (mean & SE) among men from “Bus Santé” study, Geneva, Switzerland, years 2005–2012, by socioeconomic status indicator, adjusted for age and energy.

	Education				Income				Occupation		
	Low (n = 796)	Medium (n = 539)	High (n = 1138)	*p*	Low (n = 441)	Medium (n = 898)	High (n = 1020)	*p*	Low (n = 790)	High (n = 1653)	*p*
TEI (kcal/d)	2171 (25.7)	2150 (31.4)	2080 (21.3)	0.02	2148 (34.5)	2158 (23.4)	2085 (22)	0.06	2208 (25.9)	2086 (17.5)	**
Macronutrients (g/d)											
Total proteins	81.5 (0.6)	81.9 (0.8)	83.2 (0.5)	0.10	80.8 (0.9)	81.1 (0.6)	84.1 (0.5)	**	81.3 (0.6)	82.8 (0.4)	0.06
Animal protein	57 (0.7)	57.2 (0.9)	58.1 (0.6)	0.46	55.6 (1.0)	56.4 (0.7)	59.4 (0.6)	**	56.7 (0.7)	58 (0.5)	0.15
Vegetable protein	24.5 (0.2)	24.7 (0.3)	25.1 (0.2)	0.16	25.2 (0.3)	24.7 (0.2)	24.7 (0.2)	0.41	24.7 (0.2)	24.8 (0.2)	0.55
Total carbohydrates	239 (1.8)	242 (2.2)	239 (1.5)	0.52	245 (2.4)	242 (1.6)	236 (1.5)	*	242 (1.8)	239 (1.2)	0.15
Sugars	106 (1.5)	107 (1.8)	106 (1.2)	0.85	107 (2.0)	109 (1.3)	103 (1.3)	0.01	106 (1.5)	106 (1.0)	0.79
Polysaccharides	133 (1.6)	134 (1.9)	133 (1.3)	0.75	137 (2.1)	132 (1.4)	132 (1.3)	0.07	135 (1.6)	132 (1.1)	0.17
Fibre	15.6 (0.3)	16.1 (0.3)	16.9 (0.2)	**	16.3 (0.3)	16.3 (0.2)	16.3 (0.2)	0.99	16 (0.3)	16.4 (0.2)	0.17
Total fats	81.4 (0.6)	79.1 (0.7)	82.3 (0.5)	*	80.1 (0.8)	80.4 (0.6)	82.6 (0.5)	*	79.7 (0.6)	82.1 (0.4)	*
SFA	30.5 (0.3)	29.2 (0.4)	30.8 (0.3)	**	29.7 (0.4)	29.9 (0.3)	31 (0.3)	*	29.5 (0.3)	30.8 (0.2)	**
MUFA	32.6 (0.3)	31.7 (0.4)	33.2 (0.3)	*	31.9 (0.4)	32.2 (0.3)	33.5 (0.3)	**	32 (0.3)	33 (0.2)	*
PUFA	11.7 (0.1)	11.7 (0.2)	11.4 (0.1)	0.20	11.8 (0.2)	11.6 (0.1)	11.3 (0.1)	0.07	11.8 (0.1)	11.4 (0.1)	0.03
Cholesterol (mg/d)	347 (4.5)	353 (5.4)	358 (3.7)	0.18	351 (6.0)	349 (4.1)	359 (3.8)	0.18	344 (4.5)	358 (3)	0.01
Micronutrients											
Calcium (mg/d)	1110 (17.2)	1019 (21)	1128 (14.2)	**	1046 (23.1)	1074 (15.7)	1143 (14.7)	**	1044 (17.4)	1124 (11.7)	**
Iron (mg/d)	11.6 (0.1)	11.9 (0.1)	11.9 (0.1)	*	11.5 (0.1)	11.7 (0.1)	12 (0.1)	**	11.7 (0.1)	11.9 (0.1)	0.04
Retinol (μg/d)	570 (19.3)	534 (23.5)	542 (15.9)	0.42	580 (25.8)	555 (17.5)	532 (16.4)	0.28	571 (19.4)	539 (13.1)	0.17
Carotene (μg/d)	3649 (99.1)	3604 (121)	3941 (81.9)	0.02	3630 (133)	3789 (90.3)	3824 (84.7)	0.46	3639 (100)	3838 (67.5)	0.10
Vitamin D (μg/d)	2.6 (0.1)	2.8 (0.1)	3.2 (0.1)	**	2.7 (0.1)	2.8 (0.1)	3.1 (0.1)	**	2.6 (0.1)	3 (0.1)	**

Mean and standard error of the mean from ANOVA adjusted for age and TEI; mean TEI from ANOVA adjusted for age. TEI, Total energy intake in calories per day. SE, standard error of the mean. Education: low, primary education or apprenticeship; medium, secondary education; high, tertiary education. Income: low, <5000 CHF/month (1 CHF = 1.01 USD or 0.91 EUR as of 24.02.2016); medium, 5000 to 9499 CHF/month; high, ≥9500 CHF/month. Occupation: low, blue collar; high, white collar; SFA, saturated fatty acids; MUFA, monounsaturated fatty acids; PUFA, polyunsaturated fatty acids. *p*, p-value; mean values were significantly different across groups:

*P<0.01

**P<0.001.

**Table 3:** Energy and nutrient intake (mean & SE) among women from “Bus Santé” study, Geneva, Switzerland, years 2005–2012, by socioeconomic status indicator, adjusted for age and energy.

	Education				Income				Occupation		
	Low (n = 699)	Medium (n = 893)	High (n = 1043)	*p*	Low (n = 597)	Medium (n = 998)	High (n = 782)	*p*	Low (n = 678)	High (n = 1822)	*p*
TEI (kcal/d)	1715 (24.5)	1726 (22.3)	1751 (19.5)	0.50	1720 (26.3)	1735 (19.2)	1742 (21.8)	0.82	1776 (24.7)	1719 (14.5)	0.04
Macronutrients (g/d)											
Total proteins	68.3 (0.6)	68.5 (0.6)	67.6 (0.5)	0.52	67.6 (0.7)	67.9 (0.5)	68.6 (0.6)	0.48	68.1 (0.6)	68.1 (0.4)	0.92
Animal protein	47.7 (0.7)	47.5 (0.7)	46 (0.6)	0.11	46.4 (0.8)	46.8 (0.6)	47.5 (0.7)	0.54	47 (0.7)	46.9 (0.4)	0.92
Vegetable protein	20.7 (0.2)	20.9 (0.2)	21.6 (0.2)	*	21.2 (0.3)	21.2 (0.2)	21.1 (0.2)	0.99	21.2 (0.2)	21.2 (0.1)	0.97
Total carbohydrates	202 (1.6)	201 (1.5)	201 (1.3)	0.78	206 (1.8)	202 (1.3)	197 (1.5)	**	207 (1.7)	200 (1.0)	**
Sugars	102 (1.5)	98.7 (1.4)	98.8 (1.2)	0.19	104 (1.6)	99.9 (1.2)	96.6 (1.4)	*	103 (1.6)	98.5 (0.9)	0.01
Polysaccharides	99.9 (1.5)	102 (1.4)	102 (1.2)	0.48	102 (1.6)	102 (1.2)	100 (1.3)	0.48	104 (1.5)	101 (0.9)	0.07
Fibre	15.7 (0.3)	16 (0.2)	16.8 (0.2)	*	16.2 (0.3)	16.3 (0.2)	16.2 (0.2)	0.96	16.4 (0.3)	16.2 (0.2)	0.61
Total fats	67.8 (0.6)	68.8 (0.5)	68.5 (0.5)	0.49	67 (0.6)	68.2 (0.5)	69.7 (0.5)	*	66.6 (0.6)	69 (0.3)	**
SFA	24 (0.3)	24.2 (0.2)	24 (0.2)	0.86	23.3 (0.3)	24.1 (0.2)	24.5 (0.2)	*	22.8 (0.3)	24.5 (0.2)	**
MUFA	28 (0.3)	28.6 (0.3)	28.9 (0.3)	0.11	27.6 (0.4)	28.4 (0.3)	29.4 (0.3)	**	27.7 (0.3)	28.9 (0.2)	*
PUFA	9.8 (0.1)	9.9 (0.1)	9.4 (0.1)	0.02	10 (0.1)	9.6 (0.1)	9.6 (0.1)	0.02	10 (0.1)	9.5 (0.1)	*
Cholesterol (mg/d)	290 (6.2)	291 (5.7)	298 (5.0)	0.56	296 (6.7)	297 (4.9)	288 (5.6)	0.43	285 (6.3)	296 (3.7)	0.11
Micronutrients											
Calcium (mg/d)	979.3 (15.8)	970.4 (14.4)	990.3 (12.6)	0.58	955.2 (16.9)	986.8 (12.4)	992 (14.1)	0.21	928.5 (15.9)	999.2 (9.3)	**
Iron (mg/d)	9.8 (0.1)	9.9 (0.1)	9.9 (0.1)	0.54	9.8 (0.1)	9.9 (0.1)	10 (0.1)	0.18	9.9 (0.1)	9.9 (0)	0.80
Retinol (μg/d)	498 (25.8)	440(23.5)	443 (20.5)	0.17	480 (27.7)	474 (20.2)	417 (23)	0.12	488 (26.1)	445 (15.3)	0.16
Carotene (μg/d)	4225 (154)	4622 (140)	4865 (122)	*	4654 (165)	4579 (121)	4649 (137)	0.90	4595 (155)	4629 (91.1)	0.85
Vitamin D (μg/d)	2.6 (0.1)	2.7 (0.1)	2.9 (0.1)	*	2.7 (0.1)	2.7 (0.1)	2.9 (0.1)	0.14	2.5 (0.1)	2.9 (0.1)	**

Mean and standard error of the mean from ANOVA adjusted for age and TEI; mean TEI from ANOVA adjusted for age. TEI, Total energy intake in calories per day; SE, standard error of the mean. Education: low, primary education or apprenticeship; medium, secondary education; high, tertiary education. Income: low, <5000 CHF/month (1 CHF = 1.01 USD or 0.91 EUR as of 24.02.2016); medium, 5000 to 9499 CHF/month; high, ≥9500 CHF/month. Occupation: low, blue collar; high, white collar; SFA, saturated fatty acids; MUFA, monounsaturated fatty acids; PUFA, polyunsaturated fatty acids. Mean values were significantly different across groups:

*P<0.01

**P<0.001.

Among men, significant differences between educational levels were found for fibre; total, saturated and monounsaturated fats; calcium; iron, and vitamin D. Income was positively associated with total and animal protein; total, saturated and monounsaturated fats; calcium; iron, and vitamin D, and negatively associated with total carbohydrates intake. Men of high occupation had higher intakes of total, saturated, and monounsaturated fats; calcium, and vitamin D and lower TEI (**Table 2**).

Among women, significant differences between educational levels were found for vegetable protein, fibre, carotene, and vitamin D. Income was positively associated with total, saturated, and monounsaturated fats, and negatively associated with total carbohydrates and sugar intake. Occupation was positively associated with total, saturated, monounsaturated, and polyunsaturated fats, calcium, and vitamin D, but negatively associated with total carbohydrates and sugar intakes (**Table 3**).

### Associations between SES indicators and dietary intakes, fully adjusted model

**Tables 4**and **5**summarize the associations between SES indicators and dietary intakes in the fully adjusted model for men and women, respectively.

**Table 4:** Energy and nutrient intake (mean & SE) among men from “Bus Santé” study, Geneva, Switzerland, years 2005–2012, by socioeconomic status indicator, multivariable adjusted.

	Education				Income				Occupation		
	Low (n = 796)	Medium (n = 539)	High (n = 1138)	*p*	Low (n = 441)	Medium (n = 898)	High (n = 1020)	*p*	Low (n = 790)	High (n = 1653)	*p*
TEI (kcal/d)	2153 (27.3)	2129 (32.4)	2102 (23.3)	0.42	2105 (36.4)	2141 (23.8)	2116 (23.5)	0.61	2182 (29.6)	2098 (18.6)	0.03
Macronutrients (g/d)											
Total proteins	82 (0.7)	82 (0.8)	82.8 (0.6)	0.67	80.9 (0.9)	81.1 (0.6)	84 (0.6)	*	82.1 (0.7)	82.5 (0.5)	0.73
Animal protein	57.5 (0.8)	57.3 (0.9)	57.8 (0.7)	0.89	55.6 (1)	56.4 (0.7)	59.5 (0.7)	*	57.4 (0.8)	57.7 (0.5)	0.84
Vegetable protein	24.5 (0.3)	24.7 (0.3)	25 (0.2)	0.52	25.4 (0.3)	24.8 (0.2)	24.6 (0.2)	0.17	24.7 (0.3)	24.8 (0.2)	0.78
Total carbohydrates	239 (1.9)	242 (2.2)	239 (1.6)	0.46	246 (2.5)	242 (1.6)	235 (1.6)	*	240 (2.0)	239 (1.3)	0.69
Sugars	106 (1.6)	107 (1.8)	106 (1.3)	0.71	108 (2.1)	109 (1.4)	103 (1.3)	*	106 (1.7)	106 (1.1)	0.83
Polysaccharides	133 (1.7)	134 (2)	133 (1.4)	0.83	137 (2.2)	132 (1.4)	132 (1.4)	0.16	134 (1.8)	133 (1.1)	0.55
Fibre	15.7 (0.3)	16.1 (0.3)	16.8 (0.2)	0.03	16.7 (0.4)	16.5 (0.2)	16 (0.2)	0.26	16.2 (0.3)	16.3 (0.2)	0.64
Total fats	81.6 (0.6)	79.5 (0.8)	82 (0.6)	0.03	81 (0.9)	80.6 (0.6)	82.2 (0.6)	0.14	80.6 (0.7)	81.7 (0.4)	0.24
SFA	30.6 (0.3)	29.5 (0.4)	30.7 (0.3)	0.03	30.3 (0.4)	30 (0.3)	30.8 (0.3)	0.18	30 (0.3)	30.6 (0.2)	0.18
MUFA	32.8 (0.3)	31.9 (0.4)	33 (0.3)	0.06	32.2 (0.4)	32.2 (0.3)	33.3 (0.3)	0.03	32.4 (0.4)	32.8 (0.2)	0.38
PUFA	11.6 (0.2)	11.6 (0.2)	11.5 (0.1)	0.93	11.7 (0.2)	11.6 (0.1)	11.4 (0.1)	0.43	11.6 (0.2)	11.5 (0.1)	0.49
Cholesterol (mg/d)	350 (4.7)	355 (5.6)	355 (4.1)	0.65	354 (6.3)	350 (4.1)	356 (4.1)	0.56	346 (5.1)	357 (3.2)	0.10
Micronutrients											
Calcium (mg/d)	1120 (18.1)	1039 (21.5)	1112 (15.5)	*	1083 (24.2)	1077 (15.8)	1125 (15.7)	0.10	1073 (19.7)	1111 (12.4)	0.13
Iron (mg/d)	11.7 (0.1)	11.9 (0.1)	11.9 (0.1)	0.16	11.5 (0.1)	11.8 (0.1)	12 (0.1)	*	11.8 (0.1)	11.8 (0.1)	0.53
Retinol (μg/d)	553 (20.5)	520 (24.3)	559 (17.5)	0.42	571 (27.3)	551 (17.9)	538 (17.7)	0.63	566 (22.2)	541 (14)	0.38
Carotene (μg/d)	3710 (105)	3648 (125)	3879 (89.8)	0.31	3741 (140)	3837 (91.8)	3736 (90.7)	0.70	3711(114)	3805 (71.8)	0.51
Vitamin D (μg/d)	2.6 (0.1)	2.8 (0.1)	3.1 (0.1)	**	2.8 (0.1)	2.8 (0.1)	3 (0.1)	0.19	2.9 (0.1)	2.9 (0.1)	0.57

Mean and standard error of the mean from ANOVA adjusted for age, survey year, smoking, BMI, nationality, and other SES indicators (education, income, occupation), and total energy intake. Education: low, primary education or apprenticeship; medium, secondary education; high, tertiary education. Income: low, <5000 CHF (1 CHF = 1.01 USD or 0.91 EUR as of 24.02.2016); medium, 5000 to 9499 CHF; high, ≥9500 CHF. Occupation: low, blue collar; high, white collar. SE, standard error of the mean; TEI, Total energy intake in calories per day; SFA, saturated fatty acids; MUFA, monounsaturated fatty acids; PUFA, polyunsaturated fatty acids. Mean values were significantly different across groups:

*P<0.01

**P<0.001.

**Table 5:** Energy and nutrient intake (mean & SE) among women from “Bus Santé” study, Geneva, Switzerland, years 2005–2012, by socioeconomic status indicator, multivariable adjusted.

	Education				Income				Occupation		
	Low (n = 699)	Medium (n = 893)	High (n = 1043)	*p*	Low (n = 597)	Medium (n = 998)	High (n = 782)	*p*	Low (n = 678)	High (n = 1822)	*p*
TEI (kcal/d)	1704 (25.2)	1725 (22.5)	1759 (20.4)	0.26	1715 (27.1)	1736 (19.2)	1745 (22.5)	0.71	1792 (26.6)	1714 (14.9)	0.01
Macronutrients (g/d)											
Total proteins	68.6 (0.7)	68.5 (0.6)	67.4 (0.5)	0.32	67.1 (0.7)	67.9 (0.5)	68.9 (0.6)	0.16	67.4 (0.7)	68.3 (0.4)	0.29
Animal protein	47.9 (0.7)	47.6 (0.7)	45.8 (0.6)	0.07	45.8 (0.8)	46.7 (0.6)	47.9 (0.7)	0.13	46.1 (0.8)	47.2 (0.4)	0.26
Vegetable protein	20.7 (0.2)	20.9 (0.2)	21.6 (0.2)	*	21.4 (0.3)	21.2 (0.2)	21 (0.2)	0.55	21.3 (0.3)	21.1 (0.1)	0.52
Total carbohydrates	201 (1.7)	200 (1.5)	203 (1.4)	0.44	206 (1.8)	202 (1.3)	197 (1.5)	*	206 (1.8)	200 (1.0)	*
Sugars	101 (1.6)	98.3 (1.4)	99.8 (1.3)	0.41	104 (1.7)	99.7 (1.2)	96.8 (1.4)	0.01	103 (1.7)	98.7 (0.9)	0.05
Polysaccharides	99.5 (1.5)	101.4 (1.4)	103 (1.2)	0.33	102 (1.6)	102 (1.2)	100 (1.4)	0.51	104 (1.6)	101 (0.9)	0.14
Fibre	15.7 (0.3)	16 (0.2)	16.8 (0.2)	*	16.4 (0.3)	16.3 (0.2)	16.1 (0.2)	0.65	16.5 (0.3)	16.2 (0.2)	0.33
Total fats	68.2 (0.6)	69 (0.5)	68.1 (0.5)	0.4	67.2 (0.6)	68.2 (0.5)	69.5 (0.5)	0.03	67.1 (0.6)	68.9 (0.4)	0.02
SFA	24.2 (0.3)	24.3 (0.2)	23.8 (0.2)	0.25	23.5 (0.3)	24.1 (0.2)	24.4 (0.2)	0.08	23 (0.3)	24.4 (0.2)	**
MUFA	28.2 (0.3)	28.8 (0.3)	28.6 (0.3)	0.41	27.7 (0.4)	28.5 (0.3)	29.3 (0.3)	*	28.1 (0.4)	28.7 (0.2)	0.14
PUFA	9.7 (0.1)	9.8 (0.1)	9.5 (0.1)	0.17	9.9 (0.1)	9.5 (0.1)	9.7 (0.1)	0.16	9.9 (0.1)	9.6 (0.1)	0.10
Cholesterol (mg/d)	290 (6.4)	290 (5.7)	298 (5.2)	0.58	297 (6.9)	298 (4.9)	286 (5.7)	0.28	282 (6.8)	298 (3.8)	0.04
Micronutrients											
Calcium (mg/d)	990.1 (16.2)	975.7 (14.5)	979.6 (13.1)	0.79	971.6 (17.4)	986.3 (12.4)	981.1 (14.5)	0.79	934.7 (17.1)	997.1 (9.6)	*
Iron (mg/d)	9.9 (0.1)	9.9 (0.1)	9.9 (0.1)	0.68	9.8 (0.1)	9.9 (0.1)	10 (0.1)	0.15	9.9 (0.1)	9.9 (0.0)	0.79
Retinol (μg/d)	488 (26.7)	434 (23.7)	453 (21.5)	0.30	474 (28.6)	473 (20.3)	422 (23.7)	0.23	472 (28.1)	451 (15.7)	0.52
Carotene (μg/d)	4222 (158)	4619 (141)	4870 (128)	*	4733 (170)	4593 (121)	4577 (141)	0.76	4690 (167)	4596 (93)	0.64
Vitamin D (μg/d)	2.7 (0.1)	2.7 (0.1)	2.9 (0.1)	0.29	2.8 (0.1)	2.7 (0.1)	2.8 (0.1)	0.67	2.5 (0.1)	2.9 (0.0)	*

Mean and standard error of the mean from ANOVA adjusted for age, survey year, smoking, BMI, nationality, and other SES indicators (education, income, occupation), and total energy intake. Education: low, primary education or apprenticeship; medium, secondary education; high, tertiary education. Income: low, <5000 CHF (1 CHF = 1.01 USD or 0.91 EUR as of 24.02.2016); medium, 5000 to 9499 CHF; high, ≥9500 CHF. Occupation: low, blue collar; high, white collar. SE, standard error of the mean; TEI, Total energy intake in calories per day; SFA, saturated fatty acids; MUFA, monounsaturated fatty acids; PUFA, polyunsaturated fatty acids. Mean values were significantly different across groups:

*P<0.01

**P<0.001.

Among men, significant differences between educational levels were found for calcium and vitamin D. Income was positively associated with total and animal protein and iron, and negatively associated with total carbohydrates and sugars intake. No associations were found between occupation and dietary intake (**Table 4**).

Among women, significant differences between educational levels were found for vegetable protein, fibre, and carotene. Income was positively associated with monounsaturated fat, and negatively associated with total carbohydrates intake. Occupation was positively associated with saturated fat, calcium and vitamin D, and negatively associated with total carbohydrates intake (**Table 5**).

### Sensitivity analysis

The sensitivity analysis—same models including all previously excluded participants—showed similar results. Though in the age-adjusted model for men, TEI was negatively associated with education and cholesterol was positively associated with occupation (**Table B in S1 File**), these associations became non-significant in the fully adjusted model **Table D in S1 File**). For women, sugars intake was negatively associated with income in both models (**Tables C and E in S1 File**).

## Discussion

To our knowledge, this is the first paper in Switzerland and one of the few worldwide to simultaneously assess macro and micronutrient intake differences in a population-based adult sample across the three most commonly used SES indicators [1, 19]. Our results indicate that income and education are the main drivers of SES differences in dietary intake among men; in contrast, all three indicators play a role among women. Additionally, each SES indicator seems to have an effect on specific nutrient intakes.

### Associations between SES indicators and dietary intake among men

Men with high income consumed more total and animal protein than those with low income. Few studies have assessed this: Dubois et al found a negative association in a US sample but no association in a Canadian and a French sample [19, 20]. Substantially higher meat prices in Switzerland compared to other high-income countries [21] might influence this difference. Men with high income also had higher iron intake, as previously found elsewhere [20, 22] and likely due to higher meat consumption. Men with high income also consumed smaller amounts of total carbohydrates and sugars, confirming previously reported data [19, 20, 23]. This pattern—low carbohydrate intake and high protein and fat intake—might partly be explained by higher cheese consumption among higher SES groups [24].

Men with low education consumed less vitamin D than did men with high education, as previously reported in Switzerland [13, 15] but not elsewhere [19, 20, 25, 26]. This may be due to higher fish consumption among highly educated men [13, 27], but some associations might be country-specific—Switzerland has no foods fortified with vitamin D. Unlike in our previous paper [15], we found no educational differences in MUFA and carotene, which may be because the previous paper did not mutually adjust for income and occupation, and because education was used as a dichotomous variable. Interestingly, a U-shaped association was found between education and calcium intake: men with moderate education level consumed the least calcium, unlike the previously reported lower calcium intake among men with low education [14, 20, 23]. An explanation for such association remains unclear and further studies are needed to better assess this pattern.

In the model adjusted for TEI and age, men with high occupation had higher intakes of total, saturated, and monounsaturated fats; calcium, and vitamin D and lower TEI than did men with low occupation, agreeing with previous studies [28, 29]. Conversely, in the fully adjusted model, no differences according to occupational level remained, failing to corroborate the results of a previous study which found differences by occupation in fibre, calcium and vitamin D intake after adjusting for education [13]. Still, our multivariable analyses suggest that, among men, income and education, but not occupation, are independent determinants of dietary intake.

### Associations between SES indicators and dietary intake among women

Contrary to men, all three SES indicators were independently associated with dietary intakes among women. Women with high income consumed less total carbohydrates and sugars than did those with low income, consistent with previous studies [19, 20, 23, 30]. Women with high income consumed more monounsaturated fat, as reported in a Canadian study [20].

Women with high education consumed more vegetable protein, fibre, and carotene than did women with low education, consistent with previous findings [13, 15, 19, 20, 26, 31]. The most likely explanation is higher vegetable consumption—primary sources of vegetable protein, fibre, and carotene—by women with high SES [23, 27, 32, 33]. Unlike in our previous paper [15], we found no educational differences in the intake of fiber, iron, vitamin D, and alcohol.

Women with high occupation consumed less total calories and carbohydrates than did women with low occupation, agreeing with previous findings [20, 27, 34]. This might result from differences in work requirements; those in low occupation status might exert more physical labour than those in high occupation status, thus requiring more energy consumption. Inversely, women with high occupation consumed more calcium and vitamin D, as reported elsewhere [13, 19, 20, 23]. Finally, women with high occupation consumed more saturated fat, disagreeing with previous findings [13, 20, 23]. Higher cheese consumption in high SES women might explain these patterns [24].

The SES differences in dietary intake found in our analysis might influence health inequalities, contributing to the higher prevalence of obesity, hypertension, dyslipidemia, and diabetes and to the higher risk for CVD and certain cancers [3, 4] that are consistently observed in low SES groups [5, 6]. Particularly, our findings showed that men with low income, and women with low income and occupation, consumed significantly higher total carbohydrates and sugars than did those in high SES, a behaviour that has consistently been found to be associated with the development of the aforementioned CVD risk factors and morbidities [3, 4]. Low SES men and women had lower calcium and vitamin D intakes, and low SES women had lower carotene intake, compared with those in high SES, which might translate to micronutrient deficiencies that are associated with muscle and bone weakness, cognitive impairment in older adults, and some cancers [35].

### Strengths and limitations

The main strength of our study is the joint inclusion of the three most commonly used SES indicators: education, income, and occupation. This allowed us to simultaneously assess their associations with diet in the multivariable model besides common confounders—an important procedure rarely conducted in nutritional epidemiology. The drawback of such strategy is that comparison of our findings with those from the literature should be done with caution, as most previous reports relied on a single measure of SES and failed to adjust for other SES indicators. Still, including education, income, and occupation into the report provides a clearer picture of the complex and difficult-to-ascertain impact of SES in diet. Other strengths include the high FFQ completion rate, the low attrition rate, and the constant measurement of education, income, and occupation across the study period.

This study also has some limitations worth acknowledging. First, the study sample originated exclusively in the French-speaking canton of Geneva, so the results are not applicable to the entire country, or to ages outside the 35–74 range. However, our results provide the sole assessment of the associations between SES indicators and nutrient intake in Switzerland, and could serve as reference for the other completed or ongoing Swiss studies (menu CH, CoLaus). Second, the FFQ assessed the dietary intake of the preceding four weeks only. Still, to our knowledge, there is no validated FFQ assessing annual dietary intake in Switzerland, and it has been shown that FFQs assessing dietary intake for shorter periods than one year have the same validity as FFQs assessing annual dietary intake [36]. Third, between 2005 and 2008, smaller sample sizes were randomly selected and invited to participate in the Bus Santé study because of a concurrent cohort study relying in the same infrastructure. Finally, lack of sufficiently high statistical power due to exclusion of participants with missing data might have prevented our analysis from identifying important differences in dietary intake according to SES, particularly for education and occupation among men.

### Implications for public health nutrition

Our results suggest that public health nutrition initiatives to reduce dietary—and health—inequalities require measures that go beyond education alone; among men, targeting education alone may be futile, while measures also tackling the association of income with dietary intake seem more likely to reduce inequalities (e.g. increasing purchasing power in lower SES groups). Darmon et al found that price manipulations increased social inequalities, possibly indicating that price incentives must be accompanied by education to work effectively [37]. The higher carbohydrate and sugar intake in low income participants found in our results may be reduced by increasing their purchasing power to afford more expensive foods that are low in energy and rich in nutrients; however, this would also require an understanding of which foods are energy-poor and nutrient-rich, and which foods are energy-poor and nutrient-rich. Similarly, to increase the low intake of calcium, vitamin D, and carotene among low income and low education participants in our sample, interventions should not only empower people to afford higher priced foods, but also to be able to identify which foods are rich in these micronutrients. A recent systematic review of interventions to promote healthier diets found that interventions that involved price reductions or tax incentives decreased inequalities [38]. Another review, focused exclusively on interventions at points-of-sales (supermarkets, food stores, etc.), concluded that monetary incentives showed most promise in reducing inequalities [39]. Two randomized controlled trials, in the Netherlands and New Zealand, found that price discounts had a significant impact in promoting healthier food purchasing behaviour, but education alone failed to have an impact [8, 9].

## Conclusion

In Switzerland, SES indicators—education, income, and occupation—are independently associated with differences in dietary intakes in both sexes. Among men, income and education seem to be the main determinants of dietary intake differences, while among women, all three indicators seem to play a role. To be most effective at reducing socioeconomic inequalities in diet, public health interventions should tackle the association between dietary intake and income and occupation, instead of solely focusing on education.

## Supporting information

S1 File **Figure A in S1 File. Flowchart of participants’ inclusion in statistical analysis**.
**Table A in S1 File**. **Socio-demographic characteristics and dietary intake comparison between included and excluded participants in “Bus Santé” study, Geneva, Switzerland, from 2005 to 2012**.
**Table B in S1 File. Energy and nutrient intake (mean & SE) in men from “Bus Santé” study, Geneva, Switzerland, years 2005–2012, by socioeconomic status indicator, adjusted for age and energy, including all previously excluded participants.** Mean and standard error of the mean from ANOVA adjusted for age and total energy intake; SE, standard error of the mean. Education: low, primary education or apprenticeship; medium, secondary education; high, tertiary education. Income: low, <5000 CHF (1 CHF = 1.01 USD or 0.91 EUR as of 24.02.2016); medium, 5000 to 9499 CHF; high, ≥9500 CHF. Occupation: low, blue collar; high, white collar; TEI, Total energy intake in calories per day; SFA, saturated fatty acids; MUFA, monounsaturated fatty acids; PUFA, polyunsaturated fatty acids. *P<0.01, **P<0.001. **Table C in S1 File. Energy and nutrient intake (mean & SE) in women from “Bus Santé” study, Geneva, Switzerland, years 2005–2012, by socioeconomic status indicator, adjusted for age and energy, including all previously excluded participants**. Mean and standard error of the mean from ANOVA adjusted for age and total energy intake; SE, standard error of the mean. Education: low, primary education or apprenticeship; medium, secondary education; high, tertiary education. Income: low, <5000 CHF (1 CHF = 1.01 USD or 0.91 EUR as of 24.02.2016); medium, 5000 to 9499 CHF; high, ≥9500 CHF. Occupation: low, blue collar; high, white collar; TEI, Total energy intake in calories per day; SFA, saturated fatty acids; MUFA, monounsaturated fatty acids; PUFA, polyunsaturated fatty acids. *P<0.01, **P<0.001. **Table D in S1 File. Energy and nutrient intake (mean & SE) in men from “Bus Santé” study, Geneva, Switzerland, years 2005–2012, by socioeconomic status indicator, multivariable adjusted, including all previously excluded participants.** Mean and standard error of the mean from ANOVA adjusted for age, survey year, smoking, BMI, nationality, and other SES indicators (education, income, occupation), and total energy intake; SE, standard error of the mean. Education: low, primary education or apprenticeship; medium, secondary education; high, tertiary education. Income: low, <5000 CHF (1 CHF = 1.01 USD or 0.91 EUR as of 24.02.2016); medium, 5000 to 9499 CHF; high, ≥9500 CHF. Occupation: low, blue collar; high, white collar; TEI, Total energy intake in calories per day; SFA, saturated fatty acids; MUFA, monounsaturated fatty acids; PUFA, polyunsaturated fatty acids. *P<0.01, **P<0.001. **Table E in S1 File. Energy and nutrient intake (mean & SE) in women from “Bus Santé” study, Geneva, Switzerland, years 2005–2012, by socioeconomic status indicator, multivariable adjusted, including all previously excluded participants.** Mean and standard error of the mean from ANOVA adjusted for age, survey year, smoking, BMI, nationality, and other SES indicators (education, income, occupation), and total energy intake; SE, standard error of the mean. Education: low, primary education or apprenticeship; medium, secondary education; high, tertiary education. Income: low, <5000 CHF (1 1 CHF = 1.01 USD or 0.91 EUR as of 24.02.2016); medium, 5000 to 9499 CHF; high, ≥9500 CHF. Occupation: low, blue collar; high, white collar; TEI, Total energy intake in calories per day; SFA, saturated fatty acids; MUFA, monounsaturated fatty acids; PUFA, polyunsaturated fatty acids. *P<0.01, **P<0.001.
(DOCX)
