# Supplementary material for: Independent association between socioeconomic indicators and macro- and micro-nutrient intake in Switzerland
Source: PLoS One. 2017 Apr 3;12(4):e0174578. doi: 10.1371/journal.pone.0174578 (PMC5378340; doi:10.1371/journal.pone.0174578)
Supplement: S1 File — Figure A in S1 File. Flowchart of participants’ inclusion in statistical analysis. Table A in S1 File. Socio-demographic characteristics and dietary intake comparison between included and excluded participants in “Bus Santé” study, Geneva, Switzerland, from 2005 to 2012. Table B in S1 File. Energy and nutrient intake (mean & SE) in men from “Bus Santé” study, Geneva, Switzerland, years 2005–2012, by socioeconomic status indicator, adjusted for age and energy, including all previously excluded participants. Mean and standard error of the mean from ANOVA adjusted for age and total energy intake; SE, standard error of the mean. Education: low, primary education or apprenticeship; medium, secondary education; high, tertiary education. Income: low, <5000 CHF (1 CHF = 1.01 USD or 0.91 EUR as of 24.02.2016); medium, 5000 to 9499 CHF; high, ≥9500 CHF. Occupation: low, blue collar; high, white collar; TEI, Total energy intake in calories per day; SFA, saturated fatty acids; MUFA, monounsaturated fatty acids; PUFA, polyunsaturated fatty acids. *P<0.01, **P<0.001. Table C in S1 File. Energy and nutrient intake (mean & SE) in women from “Bus Santé” study, Geneva, Switzerland, years 2005–2012, by socioeconomic status indicator, adjusted for age and energy, including all previously excluded participants. Mean and standard error of the mean from ANOVA adjusted for age and total energy intake; SE, standard error of the mean. Education: low, primary education or apprenticeship; medium, secondary education; high, tertiary education. Income: low, <5000 CHF (1 CHF = 1.01 USD or 0.91 EUR as of 24.02.2016); medium, 5000 to 9499 CHF; high, ≥9500 CHF. Occupation: low, blue collar; high, white collar; TEI, Total energy intake in calories per day; SFA, saturated fatty acids; MUFA, monounsaturated fatty acids; PUFA, polyunsaturated fatty acids. *P<0.01, **P<0.001. Table D in S1 File. Energy and nutrient intake (mean & SE) in men from “Bus Santé” study, Geneva, Switzerland, years 2005–2012, by [file pone.0174578.s001.docx]

**Table A**. Socio-demographic characteristics and dietary intake comparison between included and excluded participants in “Bus Santé” study, Geneva, Switzerland, from 2005 to 2012

|  | Included | | Excluded | |  |
| --- | --- | --- | --- | --- | --- |
|  | N | % | N | % | *p-value* |
| Total | 4429 |  | 658 |  |  |
| Age (mean years, SD) | 51.3 | 11.5 | 50.8 | 14.1 | 0.28 |
| Woman | 2157 | 48.7 | 455 | 69.2 | ** |
| BMI (kg/m^2^) | 25.5 | 9.0 | 25.3 | 13.2 | 0.68 |
| BMI categories |  |  |  |  | 0.02 |
| Underweight or normal | 2457 | 54.6 | 360 | 59.2 |  |
| Overweight | 1483 | 33.5 | 169 | 27.8 |  |
| Obese | 519 | 11.7 | 79 | 13.0 |  |
| Smoking |  |  |  |  | 0.01 |
| Never smoked | 2046 | 46.2 | 343 | 52.1 |  |
| Smoker | 957 | 21.6 | 133 | 20.2 |  |
| Ex-smoker | 1426 | 32.3 | 182 | 27.7 |  |
| Nationality |  |  |  |  | 0.05 |
| Swiss | 3096 | 69.9 | 435 | 66.1 |  |
| Other | 1333 | 30.1 | 223 | 33.9 |  |
| Education level |  |  |  |  | ** |
| Low | 1289 | 29.1 | 206 | 31.7 |  |
| Medium | 1173 | 26.5 | 229 | 35.3 |  |
| High | 1967 | 44.4 | 214 | 33.0 |  |
| Income |  |  |  |  | ** |
| Low | 913 | 20.6 | 125 | 40.7 |  |
| Medium | 1789 | 40.4 | 107 | 34.9 |  |
| High | 1729 | 39.0 | 75 | 24.4 |  |
| Occupation |  |  |  |  | ** |
| Low | 1263 | 28.5 | 205 | 39.9 |  |
| High | 3166 | 71.5 | 309 | 60.1 |  |

Number of participants (%) or mean ± standard deviation. Statistical comparison by student’s t-test for age and BMI, and chi-square for sex, smoking, BMI categories, nationality, education, income, and occupation. BMI, body max index; BMI categories, underweight or normal, BMI<25; overweight, 25≤BMI<30; obese, BMI≥30. Education: low, primary education or apprenticeship; medium, secondary education; high, tertiary education. Income: low, <5000 CHF (1 CHF = 1.01 USD or 0.91 EUR as of 24.02.2016); medium, 5000 to 9499 CHF; high, ≥9500 CHF. Occupation: low, blue collar; high, white collar.

**Figure A**. Flowchart of participants’ inclusion in statistical analysis

Total energy intake <850 or >4500 kcal/day = 185

No education or occupation data = 80

No income data = 351

No age, BMI, sex, or smoking data = 42

Total included = 4429

Total original sample = 5087

**Table B.** Energy and nutrient intake (mean & SE) in men from “Bus Santé” study, Geneva, Switzerland, years 2005-2012, by socioeconomic status indicator, adjusted for age and energy, including all previously excluded participants

|  | Education | | | | | | |  | Income | | | | | | |  | Occupation | | | | |
| --- | --- | --- | --- | --- | --- | --- | --- | --- | --- | --- | --- | --- | --- | --- | --- | --- | --- | --- | --- | --- | --- |
|  | Low | | Medium | | High | |  |  | Low | | Medium | | High | |  |  | Low | | High | |  |
|  | Mean | SE | Mean | SE | Mean | SE | *p-value* |  | Mean | SE | Mean | SE | Mean | SE | *p-value* |  | Mean | SE | Mean | SE | *p-value* |
| TEI (kcal/d) | 2172 | 25 | 2153 | 30 | 2077 | 21 | ***** |  | 2150 | 34 | 2154 | 23 | 2083 | 22 | 0.06 |  | 2209 | 25 | 2079 | 17 | ****** |
| Macronutrients (g/d) |  |  |  |  |  |  |  |  |  |  |  |  |  |  |  |  |  |  |  |  |  |
| Total proteins | 81.7 | 0.6 | 81.5 | 0.7 | 83.1 | 0.5 | 0.11 |  | 80.5 | 0.8 | 82.6 | 0.6 | 84.1 | 0.5 | ****** |  | 81.3 | 0.6 | 82.6 | 0.4 | 0.09 |
| Animal protein | 57.3 | 0.7 | 56.7 | 0.8 | 58.1 | 0.6 | 0.36 |  | 55.3 | 0.9 | 57.8 | 0.7 | 59.3 | 0.6 | ****** |  | 56.7 | 0.7 | 57.8 | 0.5 | 0.19 |
| Vegetal protein | 24.4 | 0.2 | 24.9 | 0.3 | 25.2 | 0.2 | 0.15 |  | 25.3 | 0.3 | 24.8 | 0.2 | 24.7 | 0.2 | 0.25 |  | 24.7 | 0.2 | 24.8 | 0.2 | 0.64 |
| Total carbohydrates | 239 | 2 | 244 | 2 | 239 | 1 | 0.12 |  | 246 | 2 | 239 | 2 | 236 | 2 | ****** |  | 242 | 2 | 239 | 1 | 0.14 |
| Sugars | 106 | 1 | 108 | 2 | 106 | 1 | 0.67 |  | 107 | 2 | 106 | 1 | 103 | 1 | 0.02 |  | 106 | 1 | 106 | 1 | 0.87 |
| Polysaccharides | 133 | 2 | 136 | 2 | 133 | 1 | 0.34 |  | 138 | 2 | 132 | 1 | 132 | 1 | 0.03 |  | 135 | 2 | 132 | 1 | 0.15 |
| Fiber | 15.6 | 0.3 | 16.3 | 0.3 | 16.8 | 0.2 | ***** |  | 16.4 | 0.3 | 16.4 | 0.2 | 16.3 | 0.2 | 0.87 |  | 16.4 | 0.3 | 16.4 | 0.2 | 0.17 |
| Total fats | 81.4 | 0.6 | 78.8 | 0.7 | 82.2 | 0.5 | ****** |  | 79.7 | 0.8 | 81.9 | 0.6 | 82.5 | 0.5 | ***** |  | 79.4 | 0.6 | 81.9 | 0.4 | ****** |
| SFA | 30.6 | 0.3 | 29.4 | 0.4 | 30.8 | 0.2 | ****** |  | 29.5 | 0.4 | 30.7 | 0.3 | 31.2 | 0.3 | ***** |  | 29.4 | 0.3 | 30.7 | 0.2 | ****** |
| MUFA | 32.6 | 0.3 | 31.5 | 0.4 | 33.2 | 0.3 | ***** |  | 31.8 | 0.4 | 33.2 | 0.3 | 33.4 | 0.3 | ****** |  | 31.8 | 0.3 | 33.2 | 0.2 | ***** |
| PUFA | 11.7 | 0.1 | 11.7 | 0.2 | 11.4 | 0.1 | 0.22 |  | 11.8 | 0.2 | 11.4 | 0.1 | 11.3 | 0.1 | 0.09 |  | 11.7 | 0.1 | 11.4 | 0.1 | 0.08 |
| Cholesterol (mg/d) | 347 | 4 | 350 | 5 | 359 | 4 | 0.1 |  | 349 | 6 | 358 | 4 | 358 | 4 | 0.14 |  | 342 | 4 | 358 | 3 | ***** |
| Micronutrients |  |  |  |  |  |  |  |  |  |  |  |  |  |  |  |  |  |  |  |  |  |
| Calcium (mg/d) | 1112 | 17 | 1017 | 20 | 1125 | 14 | ****** |  | 1041 | 23 | 1122 | 16 | 1141 | 15 | ****** |  | 1045 | 17 | 1122 | 12 | ****** |
| Iron (mg/d) | 11.6 | 0.1 | 11.9 | 0.1 | 11.9 | 0.1 | 0.01 |  | 11.5 | 0.1 | 11.9 | 0.1 | 12 .2 | 0.1 | ****** |  | 11.7 | 0.1 | 11.9 | 0.1 | 0.11 |
| Retinol (µg/d) | 566 | 19 | 531 | 23 | 545 | 16 | 0.45 |  | 574 | 25 | 537 | 17 | 531 | 16 | 0.31 |  | 572 | 19 | 537 | 13 | 0.13 |
| Carotene (µg/d) | 3677 | 97 | 3623 | 18 | 3951 | 81 | 0.03 |  | 3647 | 30 | 3780 | 90 | 3824 | 84 | 0.52 |  | 3672 | 98 | 3844 | 67 | 0.15 |
| Vitamin D (µg/d) | 2.6 | 0.1 | 2.7 | 0.1 | 3.2 | 0.1 | ****** |  | 2.7 | 0.1 | 2.8 | 0.1 | 3.1 | 0.1 | ****** |  | 2.6 | 0.1 | 3.0 | 0.1 | ****** |

Mean and standard error of the mean from ANOVA adjusted for age and total energy intake; SE, standard error of the mean. Education: low, primary education or apprenticeship; medium, secondary education; high, tertiary education. Income: low, <5000 CHF (1 CHF=1.01 USD or 0.91 EUR as of 24.02.2016); medium, 5000 to 9499 CHF; high, ≥9500 CHF. Occupation: low, blue collar; high, white collar; TEI, Total energy intake in calories per day; SFA, saturated fatty acids; MUFA, monounsaturated fatty acids; PUFA, polyunsaturated fatty acids. *P<0.01, **P<0.001.

**Table C.** Energy and nutrient intake (mean & SE) in women from “Bus Santé” study, Geneva, Switzerland, years 2005-2012, by socioeconomic status indicator, adjusted for age and energy, including all previously excluded participants

|  | Education | | | | | | |  | Income | | | | | | |  | Occupation | | | | |
| --- | --- | --- | --- | --- | --- | --- | --- | --- | --- | --- | --- | --- | --- | --- | --- | --- | --- | --- | --- | --- | --- |
|  | Low | | Medium | | High | |  |  | Low | | Medium | | High | |  |  | Low | | High | |  |
|  | Mean | SE | Mean | SE | Mean | SE | *p-value* |  | Mean | SE | Mean | SE | Mean | SE | *p-value* |  | Mean | SE | Mean | SE | *p-value* |
| TEI (kcal/d) | 1723 | 23 | 1728 | 20 | 1755 | 19 | 0.48 |  | 1717 | 25 | 1734 | 19 | 1741 | 22 | 0.77 |  | 1781 | 23 | 1722 | 14 | 0.03 |
| Macronutrients (g/d) |  |  |  |  |  |  |  |  |  |  |  |  |  |  |  |  |  |  |  |  |  |
| Total proteins | 68.3 | 0.6 | 68.5 | 0.5 | 68.4 | 0.5 | 0.76 |  | 67.6 | 0.7 | 68.2 | 0.5 | 68.6 | 0.6 | 0.47 |  | 68.4 | 0.6 | 68.2 | 0.4 | 0.86 |
| Animal protein | 47.6 | 0.7 | 47.5 | 0.6 | 46.4 | 0.5 | 0.27 |  | 46.5 | 0.8 | 47.1 | 0.6 | 47.5 | 0.6 | 0.52 |  | 47.2 | 0.7 | 47.1 | 0.4 | 0.86 |
| Vegetal protein | 20.7 | 0.2 | 21.1 | 0.2 | 21.6 | 0.2 | ***** |  | 21.1 | 0.2 | 21.2 | 0.2 | 21.1 | 0.2 | 0.89 |  | 21.2 | 0.2 | 21.2 | 0.1 | 0.95 |
| Total carbohydrates | 203 | 2 | 202 | 1 | 201 | 1 | 0.51 |  | 206 | 2 | 200 | 1 | 197 | 1 | ****** |  | 208 | 2 | 200 | 1 | ****** |
| Sugars | 102 | 1 | 100 | 1 | 98.2 | 2 | 0.14 |  | 104 | 2 | 98.8 | 1 | 96.1 | 1 | ***** |  | 103 | 1 | 98.8 | 1 | ***** |
| Polysaccharides | 100 | 1 | 101 | 1 | 102 | 1 | 0.67 |  | 102 | 2 | 100 | 1 | 100 | 1 | 0.45 |  | 104 | 1 | 100 | 1 | 0.04 |
| Fiber | 15.7 | 0.3 | 16.2 | 0.2 | 16.7 | 0.2 | ***** |  | 16.2 | 0.3 | 16.2 | 0.2 | 16.2 | 0.2 | 0.75 |  | 16.4 | 0.3 | 16.2 | 0.2 | 0.65 |
| Total fats | 68.2 | 0.6 | 68.5 | 0.5 | 69.1 | 0.5 | 0.42 |  | 66.8 | 0.6 | 69.2 | 0.5 | 69.8 | 0.5 | ***** |  | 66.6 | 0.6 | 69.2 | 0.3 | ****** |
| SFA | 24.2 | 0.3 | 24.2 | 0.2 | 24.1 | 0.2 | 0.92 |  | 23.3 | 0.3 | 24.5 | 0.2 | 24.6 | 0.2 | ***** |  | 22.8 | 0.3 | 24.5 | 0.2 | ****** |
| MUFA | 28.1 | 0.3 | 28.5 | 0.3 | 29.1 | 0.3 | 0.04 |  | 27.5 | 0.3 | 28.9 | 0.3 | 29.5 | 0.3 | ****** |  | 27.7 | 0.3 | 28.9 | 0.2 | ****** |
| PUFA | 9.8 | 0.1 | 9.9 | 0.1 | 9.6 | 0.1 | 0.08 |  | 10.0 | 0.1 | 9.6 | 0.1 | 9.6 | 0.1 | 0.03 |  | 10.1 | 0.1 | 9.6 | 0.1 | ***** |
| Cholesterol (mg/d) | 290 | 6 | 290 | 5 | 298 | 5 | 0.42 |  | 296 | 6 | 296 | 5 | 289 | 5 | 0.57 |  | 285 | 6 | 296 | 3 | 0.10 |
| Micronutrients |  |  |  |  |  |  |  |  |  |  |  |  |  |  |  |  |  |  |  |  |  |
| Calcium (mg/d) | 973 | 15 | 973 | 13 | 991 | 12 | 0.50 |  | 955 | 16 | 997 | 12 | 987 | 14 | 0.24 |  | 933 | 15 | 997 | 9.0 | ****** |
| Iron (mg/d) | 9.9 | 0.1 | 9.9 | 0.1 | 10.1 | 0.1 | 0.37 |  | 9.8 | 0.1 | 9.9 | 0.1 | 10.0 | 0.1 | 0.21 |  | 10.2 | 0.1 | 9.9 | 0.1 | 0.83 |
| Retinol (µg/d) | 512 | 25 | 441 | 22 | 443 | 20 | 0.06 |  | 487 | 29 | 447 | 21 | 413 | 24 | 0.05 |  | 495 | 24 | 447 | 14 | 0.09 |
| Carotene (µg/d) | 4295 | 43 | 4604 | 29 | 4872 | 17 | ***** |  | 4684 | 61 | 4607 | 20 | 4631 | 36 | 0.93 |  | 4586 | 45 | 4630 | 87 | 0.80 |
| Vitamin D (µg/d) | 2.6 | 0.1 | 2.7 | 0.1 | 3.0 | 0.1 | ****** |  | 2.7 | 0.1 | 2.9 | 0.1 | 2.9 | 0.1 | 0.10 |  | 2.5 | 0.1 | 2.9 | 0.0 | ****** |

Mean and standard error of the mean from ANOVA adjusted for age and total energy intake; SE, standard error of the mean. Education: low, primary education or apprenticeship; medium, secondary education; high, tertiary education. Income: low, <5000 CHF (1 CHF=1.01 USD or 0.91 EUR as of 24.02.2016); medium, 5000 to 9499 CHF; high, ≥9500 CHF. Occupation: low, blue collar; high, white collar; TEI, Total energy intake in calories per day; SFA, saturated fatty acids; MUFA, monounsaturated fatty acids; PUFA, polyunsaturated fatty acids. *P<0.01, **P<0.001.

**Table D.** Energy and nutrient intake (mean & SE) in men from “Bus Santé” study, Geneva, Switzerland, years 2005-2012, by socioeconomic status indicator, multivariable adjusted, including all previously excluded participants

|  | Education | | | | | | |  | Income | | | | | | |  | Occupation | | | | |
| --- | --- | --- | --- | --- | --- | --- | --- | --- | --- | --- | --- | --- | --- | --- | --- | --- | --- | --- | --- | --- | --- |
|  | Low | | Medium | | High | |  |  | Low | | Medium | | High | |  |  | Low | | High | |  |
|  | Mean | SE | Mean | SE | Mean | SE | *p-value* |  | Mean | SE | Mean | SE | Mean | SE | *p-value* |  | Mean | SE | Mean | SE | *p-value* |
| TEI (kcal/d) | 2153 | 27 | 2129 | 32 | 2102 | 23 | 0.42 |  | 2105 | 36 | 2142 | 24 | 2116 | 24 | 0.61 |  | 2182 | 30 | 2097 | 19 | 0.03 |
| Macronutrients (g/d) |  |  |  |  |  |  |  |  |  |  |  |  |  |  |  |  |  |  |  |  |  |
| Total proteins | 82 .0 | 0.7 | 82 .1 | 0.8 | 82.8 | 0.6 | 0.67 |  | 80.9 | 0.9 | 82.5 | 0.6 | 84 .4 | 0.6 | ***** |  | 82.1 | 0.7 | 82.5 | 0.5 | 0.73 |
| Animal protein | 57.5 | 0.8 | 57.3 | 0.9 | 57.8 | 0.7 | 0.89 |  | 55.6 | 1.0 | 57.7 | 0.7 | 59.5 | 0.7 | ***** |  | 57.4 | 0.8 | 57.7 | 0.5 | 0.84 |
| Vegetal protein | 24.5 | 0.3 | 24.7 | 0.3 | 25.1 | 0.2 | 0.52 |  | 25.4 | 0.3 | 24.8 | 0.2 | 24.6 | 0.2 | 0.17 |  | 24.7 | 0.3 | 24.8 | 0.2 | 0.78 |
| Total carbohydrates | 239 | 2 | 242 | 2 | 239 | 2 | 0.46 |  | 246 | 2 | 239 | 2 | 235 | 2 | ***** |  | 240 | 2 | 239 | 1 | 0.69 |
| Sugars | 105 | 2 | 107 | 2 | 106 | 1 | 0.71 |  | 108 | 2 | 106 | 1 | 103 | 1 | ***** |  | 106 | 2 | 106 | 1 | 0.83 |
| Polysaccharides | 133 | 2 | 134 | 2 | 133 | 1 | 0.83 |  | 137 | 2 | 133 | 1 | 132 | 1 | 0.16 |  | 134 | 2 | 133 | 1 | 0.55 |
| Fiber | 15.7 | 0.3 | 16.1 | 0.3 | 16.8 | 0.2 | 0.03 |  | 16.7 | 0.4 | 16.3 | 0.2 | 16.2 | 0.2 | 0.26 |  | 16.2 | 0.3 | 16.3 | 0.2 | 0.64 |
| Total fats | 81.6 | 0.6 | 79.5 | 0.8 | 82.1 | 0.6 | 0.03 |  | 81.1 | 0.9 | 81.7 | 0.6 | 82.2 | 0.6 | 0.14 |  | 80.6 | 0.7 | 81.7 | 0.4 | 0.24 |
| SFA | 30.6 | 0.3 | 29.5 | 0.4 | 30.7 | 0.3 | 0.03 |  | 30.3 | 0.4 | 30.6 | 0.3 | 30.8 | 0.3 | 0.18 |  | 30.4 | 0.3 | 30.6 | 0.2 | 0.18 |
| MUFA | 32.8 | 0.3 | 31.9 | 0.4 | 33.2 | 0.3 | 0.06 |  | 32.2 | 0.4 | 32.8 | 0.3 | 33.3 | 0.3 | 0.03 |  | 32.4 | 0.4 | 32.8 | 0.2 | 0.38 |
| PUFA | 11.6 | 0.2 | 11.6 | 0.2 | 11.5 | 0.1 | 0.93 |  | 11.7 | 0.2 | 11.5 | 0.1 | 11.4 | 0.1 | 0.43 |  | 11.6 | 0.2 | 11.5 | 0.1 | 0.49 |
| Cholesterol (mg/d) | 350 | 5 | 355 | 6 | 355 | 4 | 0.64 |  | 354 | 6 | 357 | 4 | 356 | 4 | 0.56 |  | 346 | 5 | 357 | 3 | 0.10 |
| Micronutrients |  |  |  |  |  |  |  |  |  |  |  |  |  |  |  |  |  |  |  |  |  |
| Calcium (mg/d) | 1120 | 18 | 1039 | 22 | 1112 | 16 | ***** |  | 1083 | 24 | 1111 | 16 | 1125 | 16 | 0.10 |  | 1073 | 20 | 1111 | 12 | 0.13 |
| Iron (mg/d) | 11.7 | 0.1 | 11.9 | 0.1 | 11.9 | 0.1 | 0.16 |  | 11.5 | 0.1 | 11.8 | 0.1 | 12.2 | 0.1 | ***** |  | 11.8 | 0.1 | 11.8 | 0.1 | 0.53 |
| Retinol (µg/d) | 554 | 20 | 521 | 24 | 559 | 17 | 0.42 |  | 571 | 27 | 541 | 18 | 538 | 18 | 0.63 |  | 566 | 22 | 541 | 14 | 0.38 |
| Carotene (µg/d) | 3710 | 05 | 3648 | 25 | 3879 | 90 | 0.31 |  | 3741 | 40 | 3837 | 92 | 3736 | 91 | 0.70 |  | 3711 | 14 | 3805 | 72 | 0.51 |
| Vitamin D (µg/d) | 2.6 | 0.1 | 2.8 | 0.1 | 3.1 | 0.1 | ****** |  | 2.8 | 0.1 | 2.9 | 0.1 | 3.0 | 0.1 | 0.19 |  | 2.9 | 0.1 | 2.9 | 0.1 | 0.57 |

Mean and standard error of the mean from ANOVA adjusted for age, survey year, smoking, BMI, nationality, and other SES indicators (education, income, occupation), and total energy intake; SE, standard error of the mean. Education: low, primary education or apprenticeship; medium, secondary education; high, tertiary education. Income: low, <5000 CHF (1 CHF=1.01 USD or 0.91 EUR as of 24.02.2016); medium, 5000 to 9499 CHF; high, ≥9500 CHF. Occupation: low, blue collar; high, white collar; TEI, Total energy intake in calories per day; SFA, saturated fatty acids; MUFA, monounsaturated fatty acids; PUFA, polyunsaturated fatty acids. *P<0.01, **P<0.001.

**Table E.** Energy and nutrient intake (mean & SE) in women from “Bus Santé” study, Geneva, Switzerland, years 2005-2012, by socioeconomic status indicator, multivariable adjusted, including all previously excluded participants

|  | Education | | | | | | |  | Income | | | | | | |  | Occupation | | | | |
| --- | --- | --- | --- | --- | --- | --- | --- | --- | --- | --- | --- | --- | --- | --- | --- | --- | --- | --- | --- | --- | --- |
|  | Low | | Medium | | High | |  |  | Low | | Medium | | High | |  |  | Low | | High | |  |
|  | Mean | SE | Mean | SE | Mean | SE | *p-value* |  | Mean | SE | Mean | SE | Mean | SE | *p-value* |  | Mean | SE | Mean | SE | *p-value* |
| TEI (kcal/d) | 1705 | 25 | 1725 | 22 | 1759 | 20 | 0.26 |  | 1715 | 27 | 1736 | 19 | 1745 | 22 | 0.71 |  | 1792 | 27 | 1714 | 15 | 0.01 |
| Macronutrients (g/d) |  |  |  |  |  |  |  |  |  |  |  |  |  |  |  |  |  |  |  |  |  |
| Total proteins | 68.6 | 0.7 | 68.5 | 0.6 | 67.4 | 0.5 | 0.31 |  | 67.1 | 0.7 | 68.3 | 0.5 | 68.9 | 0.6 | 0.16 |  | 67.4 | 0.7 | 68.3 | 0.4 | 0.29 |
| Animal protein | 47.9 | 0.7 | 47.6 | 0.7 | 45.8 | 0.6 | 0.07 |  | 45.8 | 0.8 | 47.2 | 0.6 | 47.9 | 0.7 | 0.13 |  | 46.1 | 0.8 | 47.2 | 0.4 | 0.26 |
| Vegetal protein | 20.7 | 0.2 | 20.9 | 0.2 | 21.6 | 0.2 | ***** |  | 21.4 | 0.3 | 21.1 | 0.2 | 21.2 | 0.2 | 0.55 |  | 21.3 | 0.3 | 21.1 | 0.1 | 0.52 |
| Total carbohydrates | 201 | 2 | 200 | 1 | 203 | 1 | 0.44 |  | 206 | 2 | 200 | 1 | 197 | 1 | ****** |  | 206 | 2 | 200 | 1 | ***** |
| Sugars | 101 | 2 | 98.2 | 1 | 100 | 1 | 0.41 |  | 104 | 2 | 99.1 | 1 | 97.2 | 1 | 0.01 |  | 103 | 2 | 99.4 | 1 | 0.05 |
| Polysaccharides | 100 | 2 | 101 | 1 | 103 | 1 | 0.32 |  | 102 | 2 | 101 | 1 | 100 | 1 | 0.51 |  | 104 | 2 | 101 | 1 | 0.14 |
| Fiber | 15.7 | 0.3 | 16.1 | 0.2 | 16.8 | 0.2 | ***** |  | 16.4 | 0.3 | 16.2 | 0.2 | 16.1 | 0.2 | 0.65 |  | 16.5 | 0.3 | 16.2 | 0.2 | 0.33 |
| Total fats | 68.2 | 0.6 | 69.3 | 0.5 | 68.1 | 0.5 | 0.40 |  | 67.2 | 0.6 | 68.9 | 0.5 | 69.5 | 0.5 | 0.03 |  | 67.1 | 0.6 | 68.9 | 0.4 | 0.01 |
| SFA | 24.2 | 0.3 | 24.3 | 0.2 | 23.8 | 0.2 | 0.25 |  | 23.5 | 0.3 | 24.4 | 0.2 | 24.4 | 0.2 | 0.08 |  | 23.2 | 0.3 | 24.4 | 0.2 | ****** |
| MUFA | 28.2 | 0.3 | 28.8 | 0.3 | 28.6 | 0.3 | 0.41 |  | 27.7 | 0.4 | 28.7 | 0.3 | 29.3 | 0.3 | ***** |  | 28.1 | 0.4 | 28.7 | 0.2 | 0.14 |
| PUFA | 9.7 | 0.1 | 9.8 | 0.1 | 9.5 | 0.1 | 0.17 |  | 9.9 | 0.1 | 9.6 | 0.1 | 9.7 | 0.1 | 0.16 |  | 9.9 | 0.1 | 9.6 | 0.1 | 0.10 |
| Cholesterol (mg/d) | 290 | 6 | 290 | 6 | 298 | 5 | 0.58 |  | 297 | 7 | 298 | 5 | 286 | 6 | 0.28 |  | 282 | 7 | 298 | 4 | 0.05 |
| Micronutrients |  |  |  |  |  |  |  |  |  |  |  |  |  |  |  |  |  |  |  |  |  |
| Calcium (mg/d) | 990 | 16 | 976 | 14 | 980 | 13 | 0.79 |  | 972 | 17 | 997 | 12 | 981 | 14 | 0.79 |  | 935 | 17 | 997 | 10 | ***** |
| Iron (mg/d) | 9.9 | 0.1 | 9.9 | 0.1 | 9.9 | 0.1 | 0.68 |  | 9.8 | 0.1 | 9.9 | 0.1 | 10.2 | 0.1 | 0.15 |  | 9.9 | 0.1 | 9.9 | 0.1 | 0.79 |
| Retinol (µg/d) | 488 | 27 | 434 | 24 | 452 | 22 | 0.30 |  | 474 | 29 | 451 | 20 | 422 | 24 | 0.23 |  | 472 | 28 | 451 | 16 | 0.52 |
| Carotene (µg/d) | 4222 | 58 | 4619 | 41 | 4870 | 28 | ***** |  | 4733 | 70 | 4593 | 21 | 4577 | 41 | 0.76 |  | 4690 | 67 | 4596 | 93 | 0.64 |
| Vitamin D (µg/d) | 2.7 | 0.1 | 2.7 | 0.1 | 2.9 | 0.1 | 0.29 |  | 2.8 | 0.1 | 2.9 | 0.1 | 2.8 | 0.1 | 0.67 |  | 2.5 | 0.1 | 2.9 | 0.1 | ***** |

Mean and standard error of the mean from ANOVA adjusted for age, survey year, smoking, BMI, nationality, and other SES indicators (education, income, occupation), and total energy intake; SE, standard error of the mean. Education: low, primary education or apprenticeship; medium, secondary education; high, tertiary education. Income: low, <5000 CHF (1 1 CHF=1.01 USD or 0.91 EUR as of 24.02.2016); medium, 5000 to 9499 CHF; high, ≥9500 CHF. Occupation: low, blue collar; high, white collar; TEI, Total energy intake in calories per day; SFA, saturated fatty acids; MUFA, monounsaturated fatty acids; PUFA, polyunsaturated fatty acids. *P<0.01, **P<0.001.
